# Pregnancy anxiety, placental corticotropin-releasing hormone and length of gestation

**DOI:** 10.1016/j.biopsycho.2022.108376

**Published:** 2022-06-03

**Authors:** Isabel F. Ramos, Kharah M. Ross, Gabrielle R. Rinne, Jennifer A. Somers, Roberta A. Mancuso, Calvin J. Hobel, Mary Coussons-Read, Christine Dunkel Schetter

**Affiliations:** a University of California, Irvine, USA; b Athabasca University, Athabasca, Alberta, Canada; c University of California, Los Angeles, USA; d Regis University, USA; e Cedars-Sinai Medical Center, Los Angeles, USA; f University of Colorado, Colorado Springs, USA

**Keywords:** Pregnancy anxiety, Length of gestation, Placental corticotrophin releasing hormone, Preterm birth, HPA-axis

## Abstract

**Objective:**

High pregnancy anxiety is a consistent predictor of earlier labor and delivery. Placental corticotropin-releasing hormone (pCRH) predicts earlier delivery consistently and it has been identified as a biological mediator of the association between pregnancy anxiety and gestational length. However, studies have not examined whether changes in pregnancy anxiety are associated with earlier birth as mediated by changes in pCRH during pregnancy. Accordingly, this study tests whether linear changes in pregnancy anxiety are associated with length of gestation indirectly through nonlinear increases in pCRH over pregnancy.

**Methods:**

A sample of pregnant women (n=233) completed prenatal assessments in early pregnancy, second trimester, and third trimester that included a 4-item assessment of pregnancy anxiety and collection of blood samples assayed for pCRH using radioimmunoassay. Length of gestation was abstracted from medical records after birth.

**Results:**

Increases in pregnancy anxiety from early pregnancy to third trimester predicted shorted length of gestation, as did nonlinear increases in pCRH over pregnancy. However, there was no evidence of an indirect effect of changes in pregnancy anxiety on length of gestation via changes in pCRH.

**Conclusions:**

These results indicate that linear changes in pregnancy anxiety and nonlinear changes in pCRH during pregnancy are independent risk factors for shortened gestational length. This study adds to a small but growing body of work on biopsychological processes in pregnancy and length of gestation. Modeling changes in psychological and biological processes during pregnancy could provide more insight into understanding risk for adverse pregnancy outcomes.

## 1. Introduction

High rates of preterm birth are a major public health concern in the United States (Purisch & Gyamfi-Bannerman, 2017) resulting in risks of maternal postpartum complications, infant mortality, developmental delays, and offspring mental health problems (Aarnoudse-Moens et al., 2009; Behrman & Butler, 2007). High pregnancy anxiety (Becker et al., 2021; Cole-Lewis et al., 2014; Goldenberg et al., 2008; Roesch et al., 2004; Ramos et al., 2019; Tomfohr-Madsen et al., 2019) and placental corticotropin-releasing hormone (pCRH) Hobel et al., 1999; McLean et al., 1995; Sandman, 2015; Wadhwa et al., 2004) consistently predict preterm birth and/or shorter length of gestation. However, studies have not tested whether changes in pregnancy anxiety are associated with changes in pCRH over pregnancy, or pCRH change as the mediational pathway.

### 1.1. Placental corticotropin-releasing hormone and pregnancy

Pregnancy can be characterized as a temporary neuroendocrine axis between the mother, fetus, and placenta. The human maternal endocrine system undergoes profound changes (O’Keane et al., 2011), with the development of the placenta over the course of pregnancy primarily responsible (Sandman, 2018). The placenta is a transient, endocrine organ, and is the first to develop, beginning to form after a fertilized egg implants in the uterus around six to seven days post conception (Haram et al., 2020). Fully formed by the third month of pregnancy (Sneddon, 2019), the placenta is a major regulator of maternal and fetal physiology, providing a direct connection between the mother and fetus (Burton & Fowden, 2015). The placenta is also a multifunctional organ, as it supplies oxygen and nutrients to the fetus, removes waste products from fetal blood, and acts as an immunological barrier between the mother and the fetus (Napso et al., 2018).

The placenta also synthesizes and secretes hormones to support the pregnancy, including placental corticotropin-releasing hormone (pCRH) (Smith et al., 2009). In the non-pregnant state, corticotropin-releasing hormone (CRH) is secreted by the hypothalamus and regulates the HPA-axis, though it is not detectable in human blood (McLean et al., 1995; Smith et al., 2009), even during extreme stress (Sandman, 2018). However, during pregnancy, the placenta expresses a gene for CRH, synthesizes, and releases substantial quantities of placental CRH (Alcántara-Alonso et al., 2017), resulting in detectable levels in maternal plasma that increase 20 to 40-fold over pregnancy (Sandman, 2018). In fact, circulating CRH in the maternal blood is nearly exclusively of placental origin (Howland et al., 2017).

Placental CRH has several roles during pregnancy. It is considered the “pregnancy clock” because it increases in maternal blood over pregnancy until it reaches a threshold that triggers labor and delivery (Ellis et al., 2002; Glover et al., 2010; Hillhouse & Grammatopoulos, 2002; Lindsay & Nieman, 2005; McLean et al., 1995; Smith & Nicholson, 2007. Several studies have shown that pCRH is involved in the regulation of human pregnancy and parturition (McLean & Smith, 2001; Hillhouse et al., 2002; Challis et al., 2000). Specifically, research indicates that pCRH acts on two CRH receptors (CRH-R1 and CRH-R2) in the myometrium during pregnancy to influence contractility (Cong et al., 2009; Zhang et al., 2008). Research in human pregnancy also reveals strong associations between levels of placental CRH and the timing of birth (Hobel et al., 1999a; Warren et al., 1992; Wolfe et al., 1987). Premature rises in pCRH levels throughout gestation have been implicated in risk for earlier gestational age and preterm birth (Hobel et al., 1999a; Sandman, 2015; Wadhwa et al., 2004).

Both trajectories and levels of pCRH at specific time points during pregnancy have been shown to predict the timing of delivery (McLean et al., 1995). Levels of pCRH in the maternal bloodstream as early as 18–20 weeks (Hobel et al., 1999b; Holzman et al., 2001; McLean et al., 1995) and as late as 30–36 weeks of gestation (Hobel et al., 1999a; Sandman et al., 2006; Smith et al., 2009; Wadhwa et al., 2004) are significantly higher in women who deliver spontaneously preterm compared to women who deliver at term. The exponential rise of pCRH throughout the course of pregnancy has been shown to be accelerated in women who experience spontaneous preterm birth (Kramer et al., 2001; McLean et al., 1995; Sandman et al., 2006; Smith et al., 2009), whereas women who deliver at term and post-term have shown slower rises in pCRH (Torricelli et al., 2006).

### 1.2. Pregnancy anxiety and length of gestation

Pregnancy anxiety is a situation-specific, negative emotional state involving worries and fears about the current pregnancy, including the health and well-being of the baby, impending childbirth, health-care experiences, and parenting (Blackmore et al., 2016; Dunkel Schetter, 2010; Guardino & Dunkel Schetter, 2014; Roesch et al., 2004). In past studies, higher pregnancy anxiety consistently predicted preterm birth and shorter length of gestation (Becker et al., 2021; Cole-Lewis et al., 2014; Goldenberg et al., 2008; Lobel & Dunkel Schetter, 2016; Lobel et al., 1992; Roesch et al., 2004; Ramos et al., 2019; Tomfohr-Madsen et al., 2019), and was a stronger predictor of preterm birth than several other psychosocial factors (Kramer et al., 2009; Lobel et al., 2008; Orr et al., 2007; Roesch et al., 2004). Two studies, however, did not find significant associations between levels of pregnancy anxiety and preterm birth or gestational length (Cole-Lewis et al., 2014; Glynn et al., 2008).

Most studies have examined the effect of pregnancy anxiety at single time points in pregnancy in the prediction of length of gestation. Evidence points to pregnancy anxiety in the second and third trimesters of pregnancy as most predictive of length of gestation (Blackmore et al., 2016; Dunkel Schetter, et al. in press Glynn et al., 2008; Kramer et al., 2009; Mancuso et al., 2004; Orr et al., 2007; Ramos et al., 2019; Rini et al., 1999; van Den Bergh, 1990; Wadhwa et al., 1993). However, these studies do not capture how anxiety about pregnancy changes, and in turn, how these changes (in pregnancy anxiety) may be related to gestational length.

Many changes take place over the course of gestation that may influence a women’s anxiety about her pregnancy. Indeed, evidence suggests that levels of pregnancy anxiety seem to be higher in the first and third trimester and lower in the second trimester (Guardino & Dunkel Schetter, 2014; Rini et al., 1999), suggesting that a woman’s anxiety is not stable over pregnancy. To date, only one study has examined whether changes in pregnancy anxiety over the course of gestation predict of length of gestation (Cole-Lewis et al., 2014) which found that changes (i.e., in pregnancy anxiety) from second to third trimester of pregnancy were significantly associated with either outcome, while second trimester levels of pregnancy anxiety were not associated with preterm birth or gestational age, and third trimester levels of pregnancy anxiety were associated with preterm birth, but not with gestational length. Thus, even though the literature indicates that levels of pregnancy anxiety are not uniform over pregnancy, it is unclear whether changes in pregnancy anxiety from early to late pregnancy are related to length of gestation.

### 1.3. Pregnancy anxiety, pCRH, and length of gestation

The activity of the maternal endocrine system during pregnancy is a potential biological mechanism through which pregnancy anxiety is hypothesized to influence the length of gestation (Mancuso et al., 2004). Maternal distress in pregnancy can accelerate pCRH production through the action of the HPA-axis end-product, cortisol (Kane et al., 2014; Hobel et al., 1999a; Thomson, 2013; Mancuso et al., 2004; Sandman, 2015). In the non-pregnant state, high levels of cortisol act as a negative feedback loop on the hypothalamus to inhibit CRH production (Sandman et al., 2018). In contrast, during pregnancy, cortisol acts on the placenta via a positive feedback loop to stimulate the synthesis and release of pCRH (Kane et al., 2014; King et al., 2001; Sandman et al., 2018).

There is evidence suggesting that higher pregnancy anxiety is associated with steeper increases in maternal cortisol trajectories (Gage et al., 2020 Kane et al., 2014; Peterson et al., 2020). However, several studies have found non-significant associations (Bleker et al., 2017; Bolten et al., 2011; Glover et al., 2010, Pluess et al., 2011, Sikkema et al., 2001). In addition, the evidence regarding cortisol and birth outcomes is mixed (Bussieres et al., 2015). However, elevated cortisol early in pregnancy has been shown to predict pCRH levels later in pregnancy (Sandman et al., 2006), and pCRH has been shown to predict length of gestation (Challis et al., 2000; Hillhouse et al., 2002; McLean & Smith, 2001; Smith et al., 1990). Thus, increases in pCRH due to maternal distress have been hypothesized to increase the risk for shorter gestation.

To date, two published studies have tested whether levels of pCRH mediate the association between pregnancy anxiety and gestational length. Mancuso et al. (2004) were the first to examine a biological marker of stress (i.e., pCRH) as a mechanism by which pregnancy anxiety influences gestational age. Although previous studies implicated prenatal stress and elevated concentrations of pCRH as reliable predictors of preterm birth, no study had examined these variables together. In this study, researchers investigated the influence of different forms of maternal stress (e.g., pregnancy anxiety, perceived stress, state-trait anxiety) and pCRH on the length of gestation (Mancuso et al., 2004). Results supported the mediation hypothesis in that levels of pCRH at 28–30 weeks gestation mediated the effect of pregnancy anxiety on the length of gestation, controlling for medical risk, income, education, and nulliparity. In other words, women with higher levels of pregnancy anxiety had shorter gestations, and higher pCRH at 28–30 weeks gestation mediated this effect. Notably, this mediational effect was found only for pregnancy anxiety, not perceived stress or state anxiety, providing evidence indicating that pregnancy anxiety may, therefore, be associated with premature increases pCRH mid-pregnancy, which in turn may increase the risk for earlier birth.

The second study to examine this mediation hypothesis tested associations between pregnancy anxiety and gestational length as mediated by pCRH levels and was the first to test linear changes in pCRH (Ramos et al., 2019). In this study, levels of pCRH in the third trimester and changes in pCRH over pregnancy predicted length of gestation. Specifically, the association between pregnancy anxiety at 19 weeks and length of gestation was mediated by pCRH at 31 weeks and by changes in pCRH from 19 to 31 weeks. These studies provide evidence that pCRH may be an HPA mechanism through which pregnancy anxiety influences gestational length (Kramer et al., 2009). However, whether changes in pregnancy anxiety are associated with changes in pCRH over the same time period as a mediational process leading to earlier delivery has never been tested.

### 1.4. The present study

In the present study, we examine changes in pregnancy anxiety from early pregnancy through third trimester in association with change in pCRH over the same period and of both with length of gestation. We theorized that change in pregnancy anxiety may accelerate pCRH trajectories from early to late pregnancy, thereby increasing risk for earlier delivery through the triggering of labor and delivery pathways. First, we tested if change in pregnancy anxiety from early to late pregnancy were associated with length of gestation. Second, we examined if change in pregnancy anxiety was associated with nonlinear change in pCRH over the same period. Third, we tested whether nonlinear change in pCRH predicted length of gestation. Finally, we tested whether linear change in pregnancy anxiety from the early pregnancy to third trimester were associated with gestational length, as mediated by nonlinear changes in pCRH over the same period. To our knowledge, this study is the first to model changes in pregnancy anxiety in relation to nonlinear changes in pCRH, and how these changes relate to length of gestation, thereby extending previous studies on pCRH as a biological mediator of the association between pregnancy anxiety and length of gestation. We hypothesized that greater increases in pregnancy anxiety would be associated with shorter length of gestation, mediated by greater increases in pCRH.

## 2. Materials and methods

### 2.1. Participants

This study used data from a prospective, longitudinal study, *Healthy Babies Before Birth* (HB3), that investigated antenatal maternal mood disorders, pregnancy anxiety, and adverse pregnancy outcomes. A complete sample description appears in Table 1. Mean maternal age at study entry was 30.33 (*SD* = 5.95) years and mean per capita income adjusted for cost of living in each of the two sites was $28,120.84 (SD = $26,749.42). More than half of participants (55.5%) completed college or earned a higher degree. Slightly over half (54.5%) the sample was pregnant with their first child, and the total number of prior pregnancies ranged from 0 to 11 (*M* = 2.17, *SD* = 1.59) and the total number of prior pregnancies resulting in a live birth ranged from 0 to 5 (*M* = 0.73, *SD* = 1.01). Most participants were either married (64.8%) or in a relationship with the baby’s father (30.2%). In terms of the ethnic and racial composition of the sample, over half of participants (61.8%) identified as not Hispanic/Latina and most participants identified as White (81.1%). The majority of women went into labor spontaneously (75.5%) and 11.2% had a scheduled C-section, with the remainder induced labor or emergency C-section. Type of labor/delivery was missing for 13.3% of the data.

### 2.2. Procedures

To be eligible for study entry a woman had to be 18 years of age or older, 12 weeks gestation, and carrying a singleton intrauterine pregnancy. Participants were excluded from the study if there was evidence of current substance abuse, HIV-positive status, smoking, or multiple gestations. Of the 301 participants who were initially enrolled into the study at T0, 233 (77.4%) women remained eligible and chose to participate at T1; these women comprised the sample for the present study. Each institution’s Institutional Review Board approved all protocols and procedures prior to study inception. Pregnant women were recruited at two urban U.S. health care sites in Los Angeles, California and Denver, Colorado. In Los Angeles, participants were recruited for data collection at a west Los Angeles major medical center serving a range of middle to higher income, mainly through direct patient contact at prenatal clinics, but also via brochures in OB/GYN practices and referral. In Denver, participants were recruited at a prenatal clinic affiliated with a major medical center serving mostly low-income women.

Trained study team staff identified pregnant women at prenatal care appointments, and if women were eligible, they were invited to participate in the study. Participants were recruited and assessed during clinic visits at the start of their pregnancy (T0) which was prior to their 16^th^ week, and at three time points during pregnancy, roughly corresponding to once per trimester: T1 at 8 to 16 weeks gestation, T2 at 20 to 26 weeks gestation, and T3 at 30 to 36 weeks gestation. Each prenatal visit included an interview, biological sample collection, and an ultrasound examination. Interviews consisted of an in-depth assessment of psychosocial constructs including pregnancy anxiety, prenatal anxiety, and maternal depression, along with many other topics. Maternal blood samples were collected at each visit, including samples for pCRH described fully below. Participants were given parking validation and $25 in cash or a gift card as compensation for each study visit.

### 2.3. Measures

#### 2.3.1. Pregnancy anxiety

Pregnancy anxiety was measured at each of the three prenatal study visits with the Pregnancy-Specific Anxiety Scale, a measure designed to assess women’s level of anxiety about their pregnancy (Roesch et al., 2004). This measure asks participants how often they experienced specific emotions due to their pregnancy in the past week. The measure consists of a list of four adjectives related to anxiety (i.e., anxious, concerned, afraid, and panicky) and eight other adjectives (i.e., lucky, excited, upset, happy, special, pleased, healthy, and in conflict – not scored). Respondents are asked to rate how often they felt these emotions on a 5-point Likert scale ranging from 1 (*never*) to 5 (*always*). Cronbach’s alpha reliabilities for the 4-item pregnancy anxiety measure were α = 0.76 at T1 and α = 0.73 at T3. Change in pregnancy anxiety was computed by subtracting T1 pregnancy anxiety from T3 pregnancy anxiety.

#### 2.3.2. Placental corticotropin-releasing hormone

Blood samples were obtained through antecubital venipuncture of women at all three prenatal assessments by nursing research staff. At each time point, the sample was collected in an aprotinin-coated vacutainer tube (BD Biosciences, San Diego, California). Immediately following collection, samples were centrifuged at 1,300–1,800 xg for 10–15 minutes at 4 °C and 1 mL of serum was harvested and stored at −80°C. Pregnancy serum samples from both sites were transported to a laboratory at the University of Colorado, Colorado Springs, for storage. Serum samples were shipped to Dr. Roger Smith’s Endocrine Lab at the University of Newcastle, Australia, as previously described (Smith et al.,2009). Samples were extracted with methanol pCRH was measured by using a radioimmunoassay. Extraction recovery was 82.5%. No correction of the data for extraction recoveries was made. The limit of sensitivity was 3 pg/mL. The intra- and inter-assay coefficients of variance (CVs) were 10.2% and 8.2%, respectively. The pCRH variable was natural log-transformed to meet assumptions of normality (West et al., 1996).

#### 2.3.3. Length of gestation

Estimated date of conception was estimated during early prenatal visits using the conventional obstetrics methods of reported last menstrual period and confirmed by pelvic ultrasound. After delivery, length of gestation and preterm birth was determined and abstracted from medical charts.

#### 2.3.4. Weeks gestation at assessment and medical history

Gestation in weeks was estimated during each prenatal visit using last menstrual period (LMP) and confirmed by pelvic ultrasound. Full information on health and pregnancy history and current pregnancy complications was coded after birth from prenatal and labor and delivery records, including relevant risk conditions and complications throughout pregnancy. This information was used to create an obstetric risk index including 6 types of risk conditions based on well-established risk factors for preterm birth (Hobel, 1982): (1) any severe infection during pregnancy or previous pregnancy; (2) hypertension during pregnancy or previous pregnancy; (3) diabetes during pregnancy or previous pregnancy; (4) any vascular risk factor, such as vaginal bleeding, anemia, placenta previa, or placental abruption; (5) oligohydramnios; and (6) polyhydramnios. Obstetric risk was treated as a continuous variable and mean centered.

Parity was assessed as a dichotomous variable representing whether or not the participant had a previous live birth (0 = prior pregnancy resulting in live birth, 1 = first-time mother). Prior history of pregnancy loss was assessed as a dichotomous variable representing whether or not the participant had a prior miscarriage or still birth (0 = no history of miscarriage or still birth, 1 = prior history of miscarriage or still birth).

#### 2.3.5. Socioeconomic status

Socioeconomic status was calculated as the sum of standardized measures of years of education completed and per capita income. Per capita income refers to total household income divided by number of persons living in the household and adjusted for cost of living in the two sites which differ^1^.

### 2.4. Data analytic plan

**Fig. 1.**
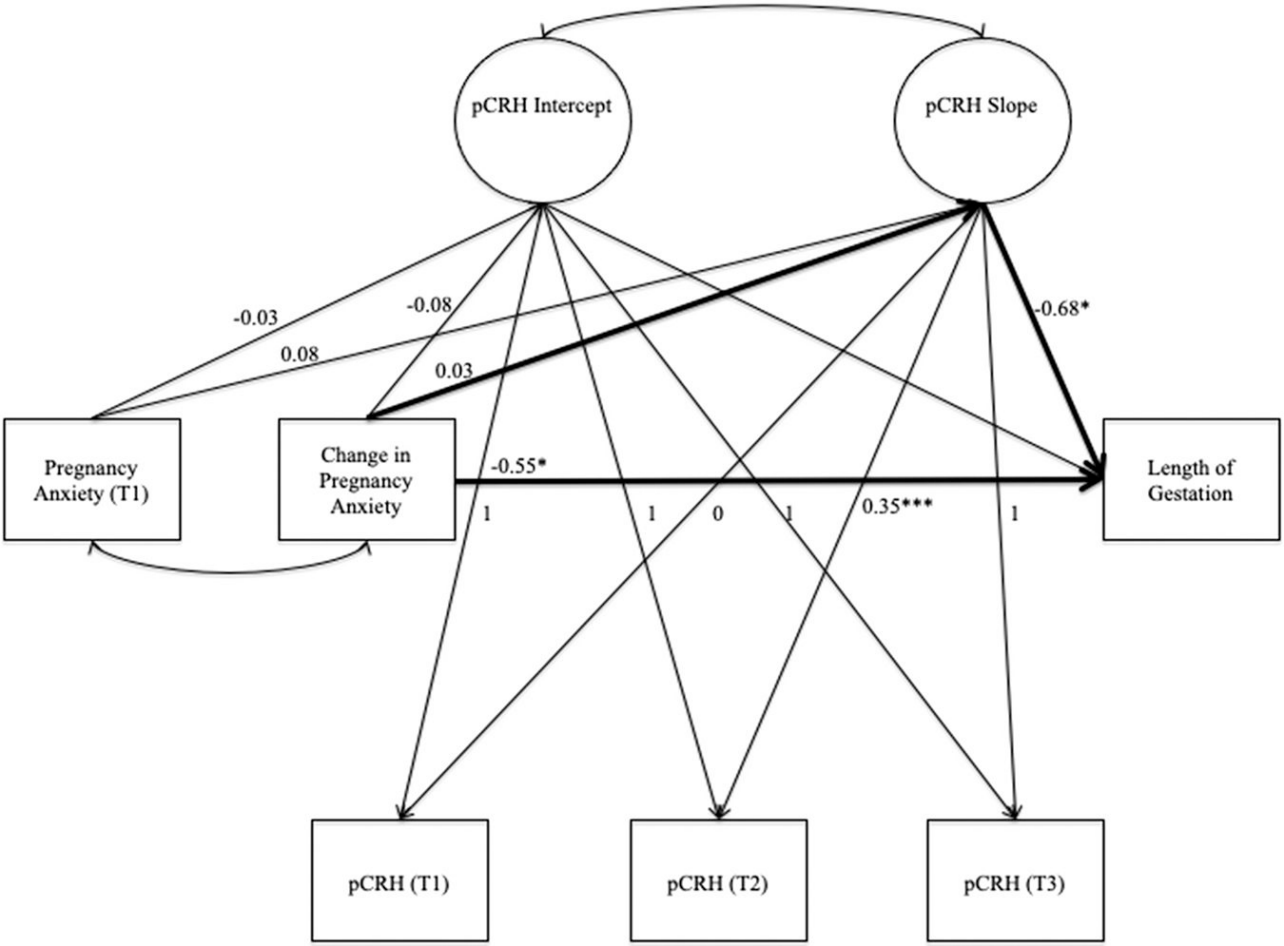
Structural equation model of the indirect effect of pregnancy anxiety on length of gestation via change in pCRH. *Note.* T1 = Early pregnancy. T2 = Second trimester. T3 = Third trimester. pCRH = placental corticotropin-releasing hormone (log-transformed). For visual clarity, covariates are not shown. * *p* < 0.05 *** *p* < 0.001.

A structural equation model was conducted to evaluate the effect of change in pregnancy anxiety on length of gestation via change in pCRH (see Fig. 1). Because pCRH increases exponentially over the course of pregnancy (McLean et al., 1995), change in pCRH was modeled with a latent basis growth curve, in which slope loadings are freely estimated to flexibly model nonlinear change (e.g., Grimm et al., 2010). In this latent basis growth curve, the intercept represents the average initial level of pCRH and the average value of the slope represents the amount of change in pCRH between the first assessment and through the third trimester. An error occurred in the initial computation of the latent basis growth curve, such that negative residual variance on T3 pCRH was estimated. Because the negative residual variance was not significantly different from 0, it was constrained to 0 in the final model.

We evaluated the indirect effect of changes in pregnancy anxiety on length of gestation via change in pCRH (shown in bold in Fig. 1) with RMediation, which produces confidence intervals (CIs) of the indirect effect based on the distribution of the product and an asymptotic normal distribution (Tofighi & MacKinnon, 2012). There is evidence for mediation if the CI for the indirect effect does not contain zero.

We conducted primary analyses using M*Plus* v.8.4 (Muthén & Muthén, 2017) using all available values and FIML. FIML uses information available from other variables and iterative optimization algorithms to estimate model parameters (Enders, 2010). FIML estimates are unbiased, more efficient than other methods of adjusting for missing data (e.g., listwise deletion), and recommended when missing data exceeds 10% (Enders & Bandalos, 2001; Little et al., 2014).

#### 2.4.1. Covariates

In the interest of parsimony, we retained the smallest set of variables that were significantly related to primary study variables in primary analyses. Early second-trimester pregnancy anxiety was included as an *a priori* covariate, in order to statistically isolate the effects of changes in pregnancy anxiety across gestation from initial levels of pregnancy anxiety. Obstetric risk (Wadhwa et al., 2001), prior history of pregnancy loss, socioeconomic status (King et al., 2001), and parity (Koullali et al., 2020) were evaluated as potential confounding variables given their possible associations with pregnancy anxiety and birth outcomes. We also evaluated weeks gestation at the time of data collection and study site (coded dichotomously) as a possible confounding variable. Only potential confounds identified from bivariate correlations that remained statistically significant in the full model were retained.

We employed bivariate correlations, independent samples t-tests, and one-way ANOVAs to evaluate whether missingness on primary study variables was related to potential confounds, and included variables related to missingness in analyses to inclusively account for missing data and to adhere to the missing at random assumption required by the estimator (Collins et al., 2001).

## 3. Results

### 3.1. Descriptive statistics

**Fig. 2.**
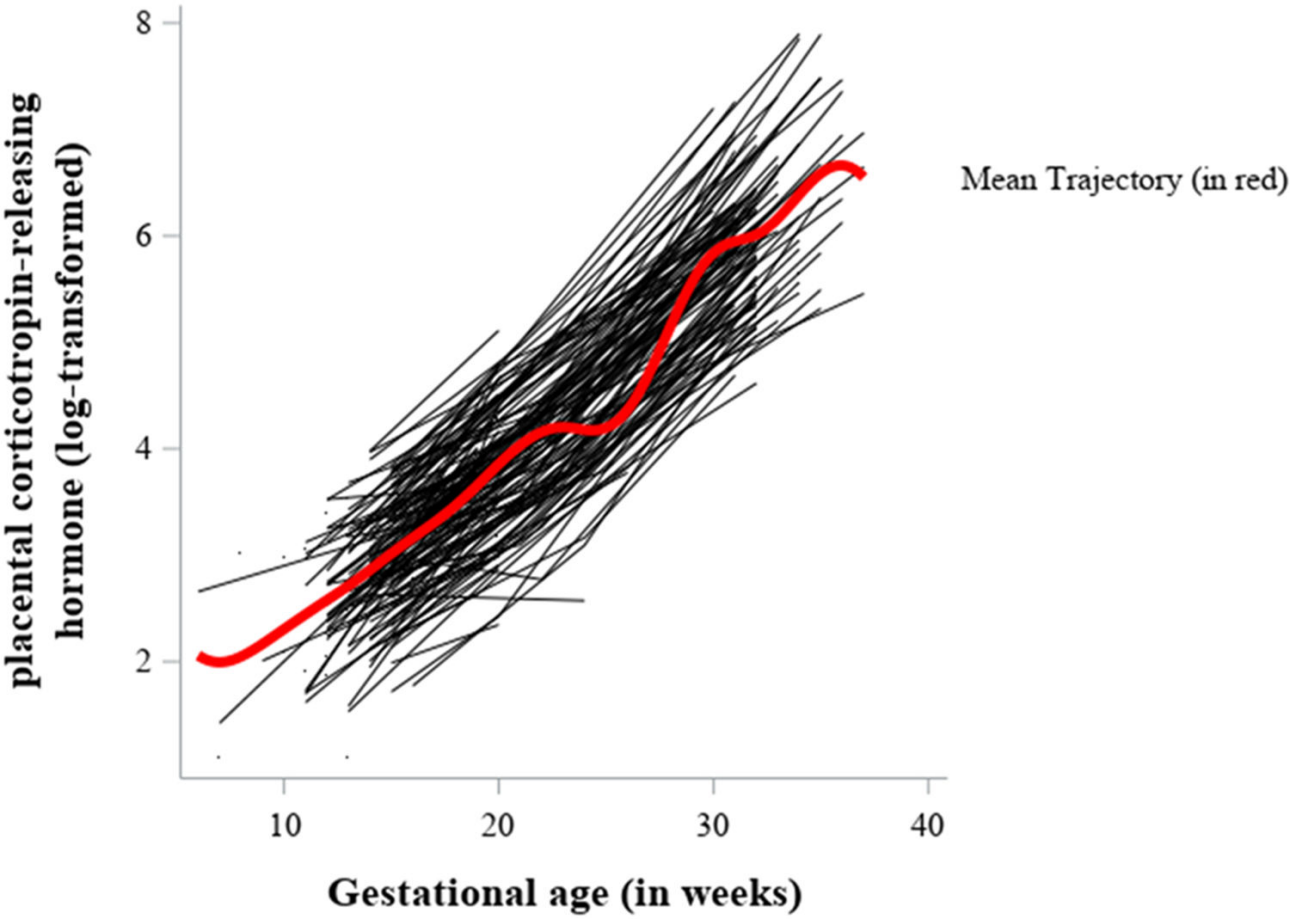
Changes in placental corticotropin-releasing hormone, by weeks gestation. Note. Black lines represent plots of changes in placental corticotropin-releasing hormone (pCRH) by weeks gestation, for each participant. The red line represents the mean penalized b-spline growth curve to illustrate the average trajectory of change in pCRH across pregnancy.

Fig. 2 illustrates change in pCRH for each participant, with a penalized b-spline superimposed to illustrate the mean trajectory of change in pCRH over the course of pregnancy. Table 2 presents descriptive statistics and bivariate correlations of T1 and T3 pregnancy anxiety, change in pregnancy anxiety, pCRH (log-transformed) at each trimester, and length of gestation. Almost half of women (49.7%) showed decreases in pregnancy anxiety, 14.1% exhibited no change in pregnancy anxiety, and 36.2% showed increases in pregnancy anxiety. Higher pregnancy anxiety at T3 was associated with shorter length of gestation. Additionally, higher levels of pCRH in early pregnancy and the second trimester were associated with longer length of gestation, but levels of pCRH in the third trimester were not associated with length of gestation.

Almost sixty percent (59.7%) of the sample did not exhibit obstetric risk, whereas 29.6% of the sample exhibited one obstetric risk factor, 4.7% of the sample exhibited two obstetric risk factors, and 0.4% of the sample exhibited three obstetric risk factors. Hypertension (14.6%) and vascular risk factors (10.7%) were the most common risk factors. Information on obstetric risk was missing for 5.6% of the sample.

There were no potential outliers (>3 *SD* from the mean) on pregnancy anxiety or on pCRH (log-transformed) at any time point. There were four potential outliers (>3 *SD* below the mean) on length of gestation.

### 3.2. Covariates

As expected, bivariate correlations indicated that changes in pregnancy anxiety were negatively related to T1 pregnancy anxiety levels, *r* = −.53, *p* <.001. Independent samples t-tests indicated that T1 pregnancy anxiety levels, *t*(225) = −2.67, *p* =.004, and changes in pregnancy anxiety, *t*(188) = 2.17, *p* =.015, differed by history of pregnancy loss, such that women who had experienced prior pregnancy loss had higher T1 pregnancy anxiety and greater decreases in pregnancy anxiety from T1 to T3. Additionally, bivariate correlations and independent samples t-tests indicated pCRH levels at T1 and T2 differed by site, socioeconomic status, obstetric risk, weeks gestation at assessment, and parity. Gestational age was negatively associated with obstetric risk (all *p*’s <.05). Thus, site, socioeconomic status, obstetric risk, history of pregnancy loss, weeks gestation at the time of assessment, and parity were evaluated as potential covariates in the final models. As noted above, in the interest of parsimony, of these potential covariates, only covariates that remained statistically significant in the final model were retained.

Of the 233 women in study sample, 229 (98.3%) had pregnancy anxiety data at T1; 194 (83.3%) had pregnancy anxiety data at T3; 174 (74.7%) had pCRH data at T1; 154 (66.1%) had pCRH data at T2; 140 (60.09%) had pCRH data at T3; and 197 (84.6%) had available length of gestation data. Obstetric risk, history of pregnancy loss, socioeconomic status, parity, site, and weeks gestation at the time of data collection were evaluated as predictors of missingness. Participants in the Denver site were more likely to be missing on T3 pCRH and gestational length, and women with lower socioeconomic status were more likely to be missing on T3 pregnancy anxiety, change in pregnancy anxiety, and gestational length (all *p*’s <.005). Participants in the Denver site were more likely to have missing medical records due to delivering in other hospitals. In order to satisfy the missing at random assumption required by the estimator, research site was obtained as a covariate of changes in pCRH and of gestational length, and socioeconomic status was retained as covariate of changes in pregnancy anxiety and gestational length in the final model.

### 3.3. Structural equation modeling results

A structural equation model tested the indirect effect of changes in pregnancy anxiety on length of gestation via change in pCRH, adjusting for control variables. We present the results of our final model with all available data because the pattern of results held when outliers were excluded. Control variables included covariates of missingness (study site and socioeconomic status) to satisfy missing at random requirements, and potential confounding variables that remained statistically significant in the final model (T1 pregnancy anxiety, weeks gestation assessed at T3, socioeconomic status, obstetric risk, and history of pregnancy loss). Because the pattern and statistical significance of the results did not differ without covariates, only the final model with covariates is presented here.

The model had acceptable fit to the data, χ^2^ (19) = 33.187, *p* = 0.02, RMSEA = 0.06 (90% CI = 0.02–0.09), CFI = 0.93, TLI = 0.85, SRMR = 0.04. Full model results are shown in Table 3. First, we report the latent basis coefficients capturing change in pCRH during pregnancy. The mean intercept was estimated as 2.670 and the mean slope was estimated as 3.385 units, representing an average increase of 126.8% in pCRH over the course of pregnancy. The freely estimated parameter for the slope loading at the second trimester was estimated at 0.354, indicating that the change between early pregnancy and the second trimester was 35.4% of the overall change between early pregnancy and the third trimester. In other words, there was a 44.9% increase between early pregnancy and the second trimester and an additional 81.9% increase between the second and third trimesters. The intercept-slope covariance was statistically significant, Est = −0.147, SE Est = 0.043, *p* = 0.001, such that women with higher initial pCRH levels showed less overall growth in pCRH over the course of pregnancy.

Regarding our primary research questions, greater increases in pregnancy anxiety were associated with shorter length of gestation, β = −0.534, SE β = 0.252, *p* = 0.034, after adjusting for the effects of early-second trimester pregnancy anxiety, research site, socioeconomic status, and weeks gestation at the third trimester research visit on length of gestation. Early-second trimester pregnancy anxiety was included as an *a priori* covariate of changes in pregnancy anxiety, and research site and socioeconomic status were included as covariates given the relation to missingness on gestational length. Socioeconomic status was also significantly related to gestational length, β = 0.203, SE β = 0.099, *p* = 0.040, as was weeks gestation, β = 0.388, SE β = 0.136, p = 0.004.

Second, changes in pregnancy anxiety did not predict increases in pCRH, β = 0.027, SE β = 0.084, *p* = 0.75, after adjusting for the effects of early-second trimester pregnancy anxiety, research site, socioeconomic status, obstetric risk, and weeks gestation at the third trimester research visit on changes in pCRH. Early-second trimester pregnancy anxiety was included as an *a priori* covariate of changes in pregnancy anxiety, and research site was included as a covariate given the relation to missingness on changes in pCRH. Socioeconomic status, β = 0.124, SE β = 0.035, *p* < 0.001; weeks gestation, β = 0.173, SE β = 0.032, *p* < 0.001; and obstetric risk, β = 0.276, SE β = 0.083, *p* = 0.001, were also retained as covariates as each were positive predictors of changes in pCRH.

Third, after adjusting for covariates, the slope of pCRH from early second to third trimester predicted length of gestation, β = −0.678, SE β = 0.322, *p* = 0.035, such that women who showed greater increases in pCRH had shorter gestational periods.

Finally, the effect of the indirect association between change in pregnancy anxiety and gestational length via change in pCRH was not significant, b = −0.018, SE = 0.064, 95% CI [−.159,.107]. This indicates that change in pregnancy anxiety was not associated with gestational length through change in pCRH.

## 4. Discussion

### 4.1. Summary of results

The purpose of this study was to build upon prior research on pregnancy anxiety and the timing of birth and to explicate further the role of placental corticotrophin hormone (pCRH) as a mediator. We tested whether changes in pregnancy anxiety between early pregnancy and third trimester were associated with gestational length, and tested mediation by changes in pCRH during the same period. Given differences among women in the changes in pregnancy anxiety over pregnancy and well-established exponential increases in pCRH, our hypotheses were evaluated with structural equation modeling testing linear change in pregnancy anxiety from early pregnancy to third trimester and nonlinear change in pCRH over the course of pregnancy. We found that greater increases in pregnancy anxiety from the early pregnancy to third trimester were associated with shorter gestational length independent of baseline pregnancy anxiety, obstetric risk, parity, and maternal sociodemographic characteristics. In addition, greater increases in pCRH from the early pregnancy to third trimester were independently associated with shorter gestational length. However, changes in pregnancy anxiety were not indirectly associated with shorter gestational length through changes in pCRH. These results add to a small but growing body of work on biopsychological processes in pregnancy and length of gestation.

### 4.2. Pregnancy anxiety as a risk factor for shortened gestational length

Pregnancy anxiety emerged as a robust predictor of gestational length in the present study. Furthermore, the effect was independent of obstetric (medical) risk factors suggesting that pregnancy complications and existing risk conditions were not responsible for effects of pregnancy anxiety on shortened length of gestation. The effect of changes in pregnancy anxiety on gestational length also held when adjusting for the associations between greater socioeconomic status (income and education) and smaller changes in pCRH with longer gestational lengths. Whether the extent of change in pregnancy anxiety during pregnancy is associated with longer gestational length had not previously been tested despite evidence that pregnancy anxiety is known to change in degree and nature over the course of gestation (Blackmore et al., 2016; Guardino & Dunkel Schetter, 2014; Rini et al., 1999; Roesch et al., 2004).

In the current study, nearly half of the women reported lower pregnancy anxiety in the third trimester relative to earlier in pregnancy, whereas a third showed higher pregnancy anxiety in third trimester, and the remainder exhibited no change. In turn, greater increases in pregnancy anxiety from early to late pregnancy were associated with shorter gestational length. These results are consistent with previous studies showing women who delivered preterm demonstrated greater increases in state anxiety between the second and third trimester (Doktorchik et al., 2018; Glynn et al., 2008). This suggests that, as with pCRH, change over time in pregnancy anxiety could be more important than absolute levels at any time in pregnancy. However, whereas the nonlinear nature of change in pCRH over pregnancy is well-characterized necessitating the use of a nonlinear model of change, research has not yet well characterized trajectories of pregnancy anxiety from early to late pregnancy partly due to the abundance of measures in use (Alderdice et al., 2012).

In a related paper from this study, four measures of anxiety including the one tested here were assessed in first and in third trimesters of pregnancy as latent factors. In each trimester, these four measures formed a latent factor and the latent factor in third trimester (but not first trimester) predicted length of gestation (Dunkel Schetter, et al. in press). The measure of pregnancy anxiety uniquely added to the prediction. The present study’s findings suggest that a brief measure to track pregnancy anxiety over the course of pregnancy may indicate women at risk for shorter gestation. The PSAS has the advantage that it is very brief and is easy to administer, however, other instruments are also available (e.g. see Blackmore et al., 2016).

### 4.3. pCRH and length of gestation

Most studies on pCRH in pregnancy have examined only associations of pCRH levels at specific times in pregnancy with gestational length (e.g., Hobel et al., 1999a; Holzman et al., 2001; McLean et al., 1995; Sandman et al., 2006). However, pCRH concentrations are known to change systematically over the course of pregnancy, with these changes regulating the timing of labor and delivery (i.e., “pregnancy clock”; McLean et al., 1995). Past studies documented associations between rate of change, and linear increases in pCRH from 19 to 31 weeks and gestational length (Ramos et al., 2019; Smith et al., 2009). However, modeling nonlinear increases in pCRH captures the exponential increases that occur over pregnancy. Indeed, we found that a greater increase in pCRH occurred between the second and third trimester compared to early pregnancy. Moreover, steeper increases in pCRH from early pregnancy to third trimester of pregnancy were associated with shorter gestation. These results are consistent with prior seminar work on the “pregnancy clock,” (McLean et al., 1995; Smith et al., 1990) and emphasize that future research must study the nonlinear nature of pCRH during pregnancy as a predictor of adverse pregnancy outcomes, especially shorter gestation length. Similarly, future work on prenatal HPA products such as maternal cortisol must examine patterns over time (Peterson et al., 2020) and ideally nonlinear patterns.

### 4.4. Mediation by pCRH

Pregnancy anxiety is thought to increase risk for adverse pregnancy outcomes by deregulating stress-related physiological processes during pregnancy, specifically by contributing to steeper increases in pCRH during later pregnancy, and thereby increasing risk for shorter gestational length (Hobel et al., 1999a; Thomson, 2013; Mancuso et al., 2004; Sandman, 2015). Two previous studies support this premise, both with larger samples (Mancuso et al., 2004; Ramos et al., 2019) that examined levels of pCRH and linear increases in pCRH support this hypothesis.

These results do not replicate earlier work on mediation of pregnancy anxiety and gestational length by levels of linear changes in pCRH. One difference is that the current study examined both changes in pregnancy anxiety and changes in pCRH in the same model to capture how pregnancy anxiety changes over pregnancy, and the exponential increases in pCRH that occur over pregnancy. Although greater increases in pregnancy anxiety and pCRH were independently associated with shorter gestation, changes in pCRH did not mediate associations between changes in pregnancy anxiety and gestation length. The smaller sample size may be a factor as prior studies involved samples of 282 and 337 women. However, these findings are consistent with results from a study of Canadian women that reported associations between higher pregnancy anxiety at 24 to 26 weeks with risk for spontaneous preterm birth, but no associations between pregnancy anxiety and pCRH in a sub-group of 117 participants (Kramer et al., 2009). Thus, detection of the biological mediation may require sufficient power. Of note, all four studies used the same 4-item measure of pregnancy anxiety.

Differences between study results on variability and mean levels in pregnancy anxiety may limit the power to detect an effect. For example, Roesch et al. (2004) and Mancuso et al. (2004) reported higher levels of pregnancy anxiety on the same measure as used here and reported in the present study.

### 4.5. Future directions

Higher pregnancy anxiety is associated with risk for more weight gain during pregnancy, smoking (Westerneng et al., 2017), poor sleep (Tomfohr-Madsen et al., 2019), and alcohol use (Arch, 2013). These health behaviors, in turn, are associated with risk for adverse birth outcomes including gestational length and/or preterm birth (Ahern et al., 2003; Huang et al., 2013; Tomfohr-Madsen et al., 2019). Pregnancy anxiety could also contribute to adverse pregnancy outcomes through additional understudied physiological pathways, including cardio-metabolic and inflammatory mechanisms. Very little research has examined associations between maternal cortisol and pCRH in pregnancy (cf. Sandman, et al. 2014). A meta-analysis suggests that maternal cortisol is not a strong predictor of birth weight or preterm birth (Bussieres et al., 2015) which contrasts with the consistent evidence that pCRH is implicated in the timing of birth. Regardless, sufficient evidence exists that pregnancy anxiety is associated with shorter gestation, and additional research is needed to elucidate the pathways and mechanisms.

We must also explore how a woman’s anxiety about her pregnancy may be influenced by her family and broader social context, and the plausible biological processes involved. We do not know whether social support from the baby’s father, the family, or the broader social network, may reduce pregnancy anxiety and its effects on physiology. Findings from one study indicate that changes in pCRH from 29 to 37 weeks gestation mediated the association between prenatal social support and lower postpartum depression (Hahn-Holbrook et al., 2013). This work emphasizes the value of testing integrated models that examine how psychosocial and biological processes involved in maternal health are intertwined.

Furthermore, women who are more anxious about their pregnancies may give birth earlier through shared decision-making processes with their physicians, leading to earlier induction of labor or scheduled C-sections, particularly in the context of high obstetric risk. This study did not focus on medical decision-making processes, and we are unable to explore this possibility. The influence of prenatal decision-making processes on pregnancy anxiety is largely unknown and should be investigated in future studies. Insomuch as the mechanisms whereby pregnancy anxiety and HPA-axis processes influence timing of birth are thought to occur mainly via spontaneous labor and delivery, this is a necessity in future studies. Finally, the sample was collected from two medical centers, one of which serves low-income women (Denver Health) and one of which serves a full range of SES including low- and middle-income women (Cedars Sinai Medical Center). Larger and more representative samples would be useful to extend this work and international research.

### 4.6. Limitations

Among this study’s limitations are the sample size that precluded the prediction of preterm birth and reduced power to test additional questions involving subgroups within the sample. In addition, studies on pCRH involve collection of blood samples from pregnant women which is time intensive and costly, often precluding its study in larger studies. Also, this study was composed of women varying in risk but studies of women at high medical risk deserve further attention. Medical risk may increase anxiety about a pregnancy though it has not accounted for much variance in past studies using various measures (see Guardino & Dunkel Schetter, 2014). However, continued attention to the risk conditions of women as they may affect pregnancy anxiety and risk of early delivery in tandem may be useful. Nonetheless, as described in Dunkel Schetter (2010), the contributors to pregnancy anxiety also include how medical risk factors are communicated, how well they are understood, a woman’s tendency to have anxiety in general, and other contextual factors in her pregnancy and her life.

Although the three prenatal assessments of pCRH allowed for modeling of nonlinear changes, additional assessment during pregnancy might better characterize the nature of change and allow for evaluation of timing and rate of change as they influence length of gestation or mediate effects of pregnancy anxiety on birth outcomes. Finally, the first prenatal visit occurred between the late first trimester and early second trimester, therefore we could not capture processes that might influence length of gestation earlier in pregnancy.

### 4.7. Conclusion

To conclude, increases in both pregnancy anxiety and pCRH from the first to third trimester of pregnancy were independently associated with shorter gestation. This is the first study to capture changes in both pregnancy anxiety and pCRH during pregnancy and relate those changes to risk for shorter gestation. However, changes in pCRH did not mediate the association between changes in pregnancy anxiety and gestational length. These findings suggest that modeling changes in psychological and biological processes during pregnancy may provide more insight into understanding risk for adverse pregnancy outcome and call for additional study.

## Supplementary Material

Supplemental Information

## Figures and Tables

**Table 1:** Sample description.

	M (SD) or %
Age at enrollment (years)	30.33 (5.95)
Per capita income ($)	28,121 (26,749)
Education level	
Less than high school	8.2
Completed high school	17.7
Some college	19.0
College or higher degree	55.5
Relationship status	
Married to baby’s father	64.8
In a relationship with baby’s father	30.2
Single	5.0
Ethnicity	
Not Hispanic/Latina	61.8
Hispanic/Latina	37.3
Race	
White	81.1
Black or African American	10.3
Asian	8.2
American Indian or Alaska Native	1.3
Native Hawaiian or Other Pacific Islander	0.4
Study Site	
Denver, Colorado	50.2
Los Angeles, California	49.8

*Note.* Per capita income adjusted for cost of living at each study site based on Cost of Living Index.

**Table 2:** Descriptive statistics and correlations.

Variable	M	SD	Range	1	2	3	4	5	6
1. T1 Pregnancy Anxiety	2.23	0.88	1-5	————					
2. T3 Pregnancy Anxiety	1.97	0.81	1-4.75	.523**	————				
3. Pregnancy Anxiety Change	−0.19	0.82	−2.25-2.25	−.534**	.441**	————			
4. T1 pCRH (log-transformed)	2.79	0.59	1.09-3.98	.029	−.038	−.124	————		
5. T2 pCRH (log-transformed)	4.02	0.61	2.35-5.41	−.037	−.111	−.103	.642**	————	
6. T3 pCRH (log-transformed)	6.14	0.69	4.22-7.90	.064	−.046	−.123	.343**	.528**	————
7. Length of gestation (weeks)	38.91	2.08	22-42	−.098	−.260**	−.025	.212*	.176*	−.028

Note.

** *p* < 0.01.

* *p* <0.05.

**Table 3:** Primary model results.

*Factor Loadings*			
Effect	Est	SE	
Early second (T1)	0	—	
Second (T2)	0.354***	0.010	
Third (T3)	1	—	
*Latent Factor Intercepts*			
Effect	Est	SE	
Intercept of pCRH	2.670***	0.059	
Slope of pCRH	3.385***	0.104	
*Beta Coefficients*			
Outcome	Predictor	Est	SE
Length of Gestation	Slope of pCRH	−0.678*	0.322
	Intercept of pCRH	0.518	0.378
	Change in pregnancy anxiety	−0.534*	0.252
	Early second (T1) pregnancy anxiety	−0.392	0.222
	Site	−0.055	0.363
	Weeks gestation at T3	0.388**	0.136
	SES	0.203*	0.099
Slope of pCRH	Change in pregnancy anxiety	0.027	0.084
	Early second (T1) pregnancy anxiety	0.078	0.084
	Site	−0.247	0.141
	Weeks gestation at T3	0.173***	0.032
	SES	0.124***	0.034
	Obstetric risk	0.276***	0.083
Intercept of pCRH	Change in pregnancy anxiety	−0.077	0.067
	Early second (T1) pregnancy anxiety	−0.030	0.061
	Site	0.277***	0.085
*Covariances*			
		Est	SE
Intercept of pCRH	Slope of pCRH	−0.147***	0.043
Change in pregnancy anxiety	Early second (T1) pregnancy anxiety	−0.396***	0.058
	Site	−0.037	0.030
	Weeks gestation at T3	0.127	0.146
	SES	−0.168	0.107
	Obstetric risk	0.009	0.035
	Prior pregnancy loss	−0.060*	0.026

*Note.* Covariances among covariates not shown for visual clarity.

*** *p* <.0001.

** *p* <.01.

* *p* <.05.

## Footnotes

1 Per capita income was divided by 1.22 for participants living in Denver or by 1.42 for participants living in Los Angeles to account for cost of living at 22% and 42% higher relative to the national average at each site, respectively.
