# Supplementary material for: Pregnancy anxiety, placental corticotropin-releasing hormone and length of gestation
Source: Biol Psychol. Author manuscript; Available in PMC 2023 Mar 17. (PMC10022399; doi:10.1016/j.biopsycho.2022.108376)
Supplement: Supplemental Information [file NIHMS1878380-supplement-Supplemental_Information.docx]

**Supplemental Information for “*Pregnancy Anxiety, Placental Corticotropin-Releasing Hormone and Length of Gestation*”**

**Supplemental Table S1.**

*Primary Model Results with Rejected Covariates*

| *Factor Loadings* | | | |
| --- | --- | --- | --- |
| Effect | Est | SE |  |
| Early second (T1) | 0 | --- |  |
| Second (T2) | 0.354*** | 0.010 |  |
| Third (T3) | 1 | --- |  |
| *Latent Factor Intercepts* | | | |
| Effect | Est | SE |  |
| Intercept of pCRH | 2.668*** | 0.059 |  |
| Slope of pCRH | 3.299*** | 0.133 |  |
| *Beta Coefficients* | | | |
| Outcome | Predictor | Est | SE |
| Length of Gestation | Slope of pCRH | -0.694* | 0.323 |
|  | Intercept of pCRH | 0.526 | 0.379 |
|  | Change in pregnancy anxiety | -0.530* | 0.251 |
|  | Early second (T1) pregnancy anxiety | -0.385 | 0.222 |
|  | Site | -0.063 | 0.363 |
|  | Weeks gestation at T3 | 0.398** | 0.134 |
|  | SES | 0.211* | 0.098 |
| Slope of pCRH | Change in pregnancy anxiety | 0.026 | 0.083 |
|  | Early second (T1) pregnancy anxiety | 0.073 | 0.083 |
|  | Site | -0.193 | 0.178 |
|  | Weeks gestation at T3 | 0.178*** | 0.032 |
|  | SES | 0.113*** | 0.036 |
|  | Obstetric risk | 0.271*** | 0.083 |
|  | Weeks gestation at T2 | -0.020 | 0.040 |
|  | First-time mother | 0.097 | 0.106 |
| Intercept of pCRH | Change in pregnancy anxiety | -0.076 | 0.067 |
|  | Early second (T1) pregnancy anxiety | -0.029 | 0.061 |
|  | Site | 0.281*** | 0.085 |
| *Covariances* | | | |
|  |  | Est | SE |
| Intercept of pCRH | Slope of pCRH | -0.144*** | 0.043 |
| Change in pregnancy anxiety | Early second (T1) pregnancy anxiety | -0.398*** | 0.059 |
|  | Site | -0.041 | 0.030 |
|  | Weeks gestation at T3 | 0.117 | 0.147 |
|  | SES | -0.193 | 0.108 |
|  | Obstetric risk | 0.008 | 0.035 |
|  | Prior pregnancy loss | -0.068* | 0.027 |
|  | Weeks gestation at T2 | -0.079 | 0.099 |
|  | First-time mother | -0.038 | 0.030 |

*Note*. X2(28) = 59.08, *p* = .0005; RMSEA = 0.069 (90% CI: 0.044-0.094); CFI = 0.86; TLI = 0.744; SRMR = 0.052.

Covariances among covariates not shown for visual clarity.

*** *p* < .0001. ** *p* < .01. * *p* < .05.
